# Radical prostatectomy versus radiotherapy as local therapy for primary tumors in patients with oligometastatic prostate cancer

**DOI:** 10.3389/fonc.2024.1368926

**Published:** 2024-03-13

**Authors:** Won Sik Ham, Jee Soo Park, Won Sik Jang, Jongchan Kim

**Affiliations:** ^1^ Department of Urology and Urological Science Institute, Yonsei University College of Medicine, Seoul, Republic of Korea; ^2^ Department of Urology, Yongin Severance Hospital, Yonsei University Health System, Yongin, Republic of Korea

**Keywords:** prostate cancer, oligometastasis, local therapy, radiotherapy, prostatectomy

## Abstract

**Introduction:**

We compared radical prostatectomy (RP) and radiotherapy (RT) as local therapies for primary tumors and examined their associations with survival outcomes and urinary tract complications in patients with oligometastatic prostate cancer (omPC).

**Methods:**

We evaluated the data of 85 patients diagnosed with omPC who underwent local therapy for primary tumors between January 2008 and December 2018. Of the 85 patients, 31 underwent prostate RT, while 54 underwent RP. Oligometastatic disease was defined as the presence of fewer than five metastatic lesions without visceral metastasis. Urinary tract complications, progression-free survival (PFS), cancer-specific survival (CSS), and overall survival (OS) were evaluated using the Kaplan–Meier method and Cox regression analyses.

**Results:**

Patients treated with RT showed higher prostate-specific antigen levels. There was no significant difference in the 5-year PFS (52.5% vs. 37.9%, p=0.351), CSS (67.6% vs. 84.7%, p=0.473), or OS (63.6% vs. 73.8%, p=0.897) between the RT and RP groups. In the multivariate analyses, the type of local therapy was not associated with PFS (hazard ratio [HR]=1.334, p=0.356), CSS (HR=0.744, p=0.475), or OS (HR=0.953, p=0.897).

**Conclusion:**

Therefore, RP seems to be a possible treatment option for patients with omPC, exhibiting oncologic outcomes comparable to those with RT.

## Introduction

Since the prostate-specific antigen (PSA) has been used to screen for prostate cancer (PC), the proportion of patients with advanced PC at the time of diagnosis has decreased. Approximately 1.6% of patients diagnosed with PC between 1998 and 2003 had metastatic lesions at the time of diagnosis (1). Androgen deprivation therapy (ADT) has been the standard treatment modality for metastatic PC (mPC) for several decades (2). In recent years, various attempts have been made to improve the outcomes of patients with mPC, and several systemic agents, such as docetaxel, abiraterone, and apalutamide, are currently recommended for the treatment of mPC according to guidelines (3, 4). However, radical prostatectomy (RP) and radiotherapy (RT) can be used to treat clinically localized disease with a curative aim (5).

Several studies have reported that local therapy for primary tumors in patients with mPC can improve survival (6–9). Some of these studies included patients with limited metastatic lesions, which could be classified as oligometastasis (7, 8). Oligometastasis refers to a limited number of metastatic lesions and/or sites. Tumors in the early stages of progression may exhibit metastasis that are limited in number and location due to the incomplete development of metastatic growth capability and restricted growth sites (10). Consequently, local therapy for the primary tumor or metastasis-directed therapy could be performed with curative intent in patients with oligometastatic cancer (11).

Studies have shown that treating primary cancer with RT improves some oncologic outcomes in patients diagnosed with oligometastatic prostate cancer (omPC) (12–14). Moreover, the National Comprehensive Cancer Network guidelines suggest that RT, in addition to ADT, is a treatment option for patients having mPC with a low metastatic burden. Some studies have compared RP to RT in PC, and reported the former to be noninferior (15, 16). However, studies comparing the results between RP and RT in omPC are extremely rare. Hence, we compared the survival outcomes of patients with omPC treated with RP or RT as a local therapy for primary tumors. We also aimed to evaluate whether there was a difference in the rate of urinary tract complications caused by disease progression between these two groups.

## Methods

### Patient selection and data collection

In this retrospective study, we reviewed the records of patients with mPC who underwent RP or RT as a local therapy for primary tumors between January 2008 and December 2018. Oligometastatic disease was defined as the presence of fewer than five metastatic lesions without visceral metastasis on preoperative imaging evaluation, including abdominopelvic computed tomography (CT), chest CT, whole-body positron emission tomography (PET)-CT, and bone scans (8). Nonregional lymph node metastasis were not classified as visceral metastasis. After excluding patients with a gap of >3 months from biopsy to local therapy and those with insufficient clinical information, 85 patients were enrolled in the final analysis. Of the 85 patients, 54 underwent RP and 31 received RT. All patients underwent ADT after being diagnosed with mPC. Treatment after local therapy was based on the physician’s clinical judgment after a discussion with the patient regarding the probable benefits and adverse effects of each treatment.

Clinicopathological characteristics, including age, body mass index (BMI), initial PSA level, Charlson Comorbidity Index (CCI), biopsy Gleason grade (GG), and clinical stage, were obtained through a review of medical records and preoperative imaging. Stages were determined according to the guidelines of the 8^th^ edition of the *American Joint Committee on Cancer Staging Manual* (17). Calculating CCI has been previously introduced in various studies (18). Among the patients who underwent RP, 12 received open RP, while 42 received robot-assisted RP; among those who received RT, 6 received three-dimensional conformal RT and 25 received intensity-modulated external beam RT. The median RT dose was 64.0 Gy (IQR=60.0–70.0 Gy), with a median fraction of 25 (IQR=24–28 Gy).

We additionally investigated urinary tract complications, defined as problems in the urinary tract due to disease progression. Only cases that required surgical intervention were included in our analysis.

### Follow-up and endpoints

After RP or RT, PSA levels were monitored every 1–3 months during the 2^nd^ year and semiannually thereafter. If there was an increase in the PSA level or if a patient developed symptoms that appeared to be caused by disease progression, imaging studies such as bone scans or abdominal/pelvic CT were performed. Treatment decisions for patients with progressive disease, such as chemotherapy or palliative RT, were made based on the physician’s clinical judgment after discussion with the patient according to the disease status and the patient’s general condition.

We compared the progression-free survival (PFS), overall survival (OS), and cancer-specific survival (CSS) between the two groups. Information on survival status and cause of death was obtained from the medical records in the Cancer Registry Center database at our institution. PFS was defined as the time from the diagnosis to disease progression, relapse on follow-up imaging, or death from any cause. CSS was defined as the time from PC diagnosis to death. OS was defined as the period from PC diagnosis to death from any cause.

### Statistical analysis

We compared the clinicopathological characteristics and urinary tract complications between the groups using the Mann–Whitney *U* test for continuous data and the chi-square test or Fisher’s exact test for dichotomous variables. We used the Kaplan–Meier method with log-rank tests to estimate and compare the PFS, CSS, and OS values between the two groups. Cox proportional hazard models were used to investigate the associations between the variables and the risk of survival outcomes. Comparisons with p<0.05 were considered statistically significant. All analyses were performed using STATA^®^ version 15.1 (StataCorp LLC, College Station, TX, USA).

### Good clinical practice protocols

This study was performed in accordance with the applicable laws and regulations, good clinical practice, and ethical principles described in the Declaration of Helsinki. The study protocol was approved by the Institutional Review Board (IRB) of Yongin Severance Hospital (approval number: 9-2022-0082). Due to the retrospective nature of the study, the IRB of Yongin Severance Hospital waived the requirement for obtaining informed consent.

## Results

### Patient characteristics

**Table 1** shows the demographic characteristics of the patients. The median initial PSA value was higher in the RT group than in the RP group (79.7 vs 32.5 ng/mL, p=0.024); however, there were no significant differences observed in age, BMI, CCI, biopsy GG, or clinical stage between the two groups. Pelvic lymph node dissection was conducted in 34 patients (63.0%) in the RP group, while radiation to lymph nodes was administered in all 31 patients in the RT group (100.0%).

**Table 1:** Patient characteristics.

Characteristics	RT	RP	p-value
	31	54	
Age (median, IQR)	70 (65–75)	65.5 (62–70.25)	0.232
BMI (median, IQR)	23.2 (22.4–26.2)	24.0 (22.68–25.5)	0.959
PSA (median, IQR)	79.7 (41.05–177.8)	32.45 (15.2–60.3)	0.024
CCI			0.910
≤1	25 (80.6%)	43 (79.6%)	
≥2	6 (19.4%)	11 (20.4%)	
Biopsy GG			0.536
≤4	18 (58.1%)	35 (64.8%)	
5	13 (41.9%)	19 (35.2%)	
Clinical T stage			0.949
≤T3	26 (83.9%)	45 (83.3$)	
T4	5 (16.1%)	9 (16.7%)	
Clinical N stage			0.886
N0	20 (64.5)	34 (63.0%)	
N1	11 (35.5%)	20 (37.0%)	
Clinical M stage			0.718
M1a	4 (12.9%)	5 (9.3%)	
M1b	27 (87.1%)	49 (90.7%)	
Pelvic LN treatment	31 (100%)	34 (63.0%)	<0.001

RP, radical prostatectomy; RT, radiotherapy; IQR, interquartile range; BMI, body mass index; PSA, prostate-specific antigen; CCI, Charlson comorbidity index; GG, Gleason grade; LN, lymph node.

### Oncologic outcomes

**Figure 1:**
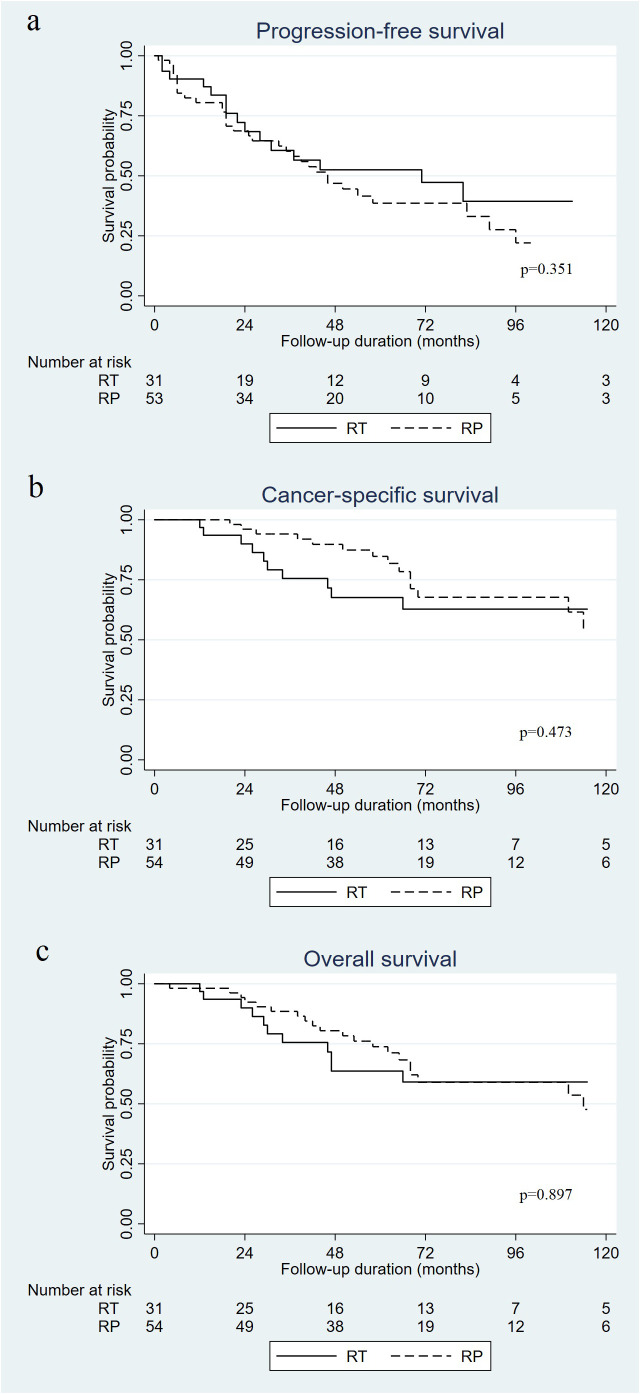
Kaplan–Meier curves of progression-free survival **(A)**, cancer specific survival **(B)**, and overall survival **(C)** in patients who received radical prostatectomy (RP) versus radiotherapy (RT) for oligometastatic prostate cancer.

At a median follow-up of 62 months (interquartile range [IQR]: 39–83.5 months), 32 patients had died (25 due to PC). The 5-year PFS rate was 52.5% in the RT group and 37.9% in the RP group, and the difference was not significant (p=0.351) (**Figure 1A**). There were no significant differences in CSS (**Figure 1B**; 67.6% vs. 84.7%, p=0.473) or OS (**Figure 1C**; 63.6% vs. 73.8%, p=0.897) between the RT and RP groups.

Multivariate analysis showed that PSA level (hazard ratio [HR]=1.002, 95% confidence interval [CI]=1.001–1.002, p=0.03) and biopsy GG (HR=2.504, 95% CI=1.384–4.531, p=0.002) were associated with PFS (**Table 2**). Regarding CSS, age (HR=1.110, 95% CI= 1.031–1.195, p=0.006), PSA level (HR=1.004, 95% CI=1.001–1.007, p=0.011), biopsy GG (HR=2.792, 95% CI=1.197–6.515, p=0.018), and clinical N stage (HR=3.564, 95% CI=1.521–8.351, p=0.003) were associated with worse CSS in the multivariate analysis (**Table 3**). The factors associated with OS were age (HR=1.094, 95% CI=1.026–1.165, p=0.006) and higher biopsy GG (HR=2.383, 95% CI=1.163–4.881, p=0.018) (**Table 4**). Whether patients received RP or RT was not associated with PFS (HR=1.334, 95% CI=0.724–2.459, p=0.356), CSS (HR=0.744, 95% CI=0.330–1.677, p=0.475), or OS (HR= 0.953, 95% CI=0.456–1.990, p=0.897).

**Table 2:** Cox proportional hazard regression analysis of factors associated with progression-free survival.

Variables	Univariate analysis		Multivariate analysis	
	HR (95% CI)	p-value	HR (95% CI)	p-value
Age	0.990 (0.945–1.037)	0.664		
BMI	1.007 (0.893–1.136)	0.904		
PSA	1.003 (1.000–1.005)	0.024	1.002 (1.000–1.004)	0.03
CCI		0.476		
≤1	1 (Ref)			
≥2	1.291 (0.640–2.602)			
Biopsy GG		0.002		0.002
≤4	1 (Ref)		1 (Ref)	
5	2.535 (1.404–4.575)		2.504 (1.384–4.531)	
Clinical T stage		0.239		
≤T3	1 (Ref)			
T4	1.500 (0.764–2.945)			
Clinical N stage		0.092		
N0	1 (Ref)			
N1	1.635 (0.923–2.896)			
Clinical M stage		0.587		
M1a	1 (Ref)			
M1b	1.330 (0.475–3.722)			
Pelvic LN treatment	1.330 (0.642–2.755)	0.443		
Type of local therapy		0.356		
RP	1 (Ref)			
RT	1.334 (0.724–2.459)			

HR, hazard ratio; CI, confidence interval; BMI, body mass index; PSA, prostate-specific antigen; CCI, Charlson comorbidity index; GG, Gleason grade; RP, radical prostatectomy; RT, radiotherapy; LN, lymph node.

**Table 3:** Cox proportional hazard regression analysis of factors associated with cancer-specific survival.

Variables	Univariate analysis		Multivariate analysis	
	HR (95% CI)	p-value	HR (95% CI)	p-value
Age	1.080 (1.007–1.158)	0.03	1.110 (1.031–1.195)	0.006
BMI	0.890 (0.755–1.048)	0.163		
PSA	1.004 (1.001–1.006)	0.009	1.004 (1.001–1.007)	0.011
CCI		0.474		
≤1	1 (Ref)			
≥2	1.436 (0.533–3.871)			
Biopsy GG		0.038		0.018
≤4	1 (Ref)		1 (Ref)	
5	2.350 (1.047–5.277)		2.792 (1.197–6.515)	
Clinical T stage		0.209		
≤T3	1 (Ref)			
T4	1.811 (0.718–4.569)			
Clinical N stage		0.015		0.003
N0	1 (Ref)		1 (Ref)	
N1	2.802 (1.224–6.417)		3.564 (1.521–8.351)	
Clinical M stage		0.072		
M0	1 (Ref)			
M1	24.07 (0.082–7045)			
Pelvic LN treatment	5.666 (0.762–42.139)	0.090		
Type of local therapy		0.475		
RP	1 (Ref)			
RT	0.744 (0.330–1.677)			

HR, hazard ratio; CI, confidence interval; BMI, body mass index; PSA, prostate-specific antigen; CCI, Charlson comorbidity index; GG, Gleason grade; RP, radical prostatectomy; RT, radiotherapy; LN, lymph node.

**Table 4:** Univariate and multivariate analysis of factors associated with overall survival.

Variables	Univariate analysis		Multivariate analysis	
	HR (95% CI)	p-value	HR (95% CI)	p-value
Age	1.085 (1.021–1.154)	0.009	1.094 (1.026–1.165)	0.006
BMI	0.909 (0.787–1.050)	0.194		
PSA	1.003 (1.000–1.005)	0.063		
CCI		0.05		0.298
≤1	1 (Ref)		1 (Ref)	
≥2	2.185 (1.001–4.771)		1.579 (0.668–3.736)	
Biopsy GG		0.041		0.018
≤4	1 (Ref)		1 (Ref)	
5	2.095 (1.031–4.254)		2.383 (1.163–4.881)	
Clinical T stage		0.56		
≤T3	1 (Ref)			
T4	1.304 (0.534–3.181)			
Clinical N stage		0.183		
N0	1 (Ref)			
N1	1.617 (0.798–3.276)			
Clinical M stage		0.191		
M0	1 (Ref)			
M1	3.781 (0.515–27.77)			
Pelvis LN treatment	1.709 (0.595–4.911)	0.320		
Type of local therapy		0.897		
RP	1 (Ref)			
RT	0.953 (0.456–1.990)			

HR, hazard ratio; CI, confidence interval; BMI, body mass index; PSA, prostate-specific antigen; CCI, Charlson comorbidity index; GG, Gleason grade; RP, radical prostatectomy; RT, radiotherapy; LN, lymph node.

### Urinary tract complications

The urinary tract complications requiring intervention in each group are shown in **Table 5**. Notably, while the rate of complications requiring surgical intervention was significantly higher in the RT group than that in the RP group (12.9% vs. 1.9%), the difference did not reach statistical significance (p=0.057). In the RP group, one patient underwent double-J stent insertion. Two patients in the RT group developed obstructive uropathy; therefore, double-J stent indwelling and percutaneous nephrostomy were required. Additionally, one patient underwent cystoscopic cauterization due to hematuria, while another required Foley catheterization.

**Table 5:** Urinary tract complications requiring a surgical procedure.

Complication and procedure	RT	RP	p-value
	31	54	
Total	4 (12.9%)	1 (1.9%)	0.057
Hematuria			
Cystoscopic cauterization	1 (3.2%)		
Urinary retention			
Foley catheterization	1 (3.2%)		
Obstructive uropathy			
Double J stent indwelling	1 (3.2%)	1 (1.9%)	
Percutaneous nephrostomy	1 (3.2%)		

RP, radical prostatectomy; RT, radiotherapy.

## Discussion

The concept of oligometastasis was first described in 1995, referring to limited metastatic lesions, which are considered an intermediate state between localized disease and widespread metastasis (10). Local therapy for the primary tumor or metastasis-directed therapy can be performed with curative intent in select patients with limited metastatic lesions (11). However, despite advancements in research, the definition of omPC remains unclear. For instance, Soloway et al. (19). assessed the relationship between the extent of metastatic lesions and mortality, dividing patients into four groups based on disease extent. The authors reported a 2-year OS rate of 96% in patients with fewer than six metastatic lesions on their bone scans, which was significantly higher than that observed in other groups having more extensive metastatic lesions. Furthermore, previous studies on omPC have shown diverse adoption of the term’s definition, which remains ambiguous but generally includes a maximum of three to five metastasis. Therefore, based on these studies, we defined oligometastasis as the presence of fewer than five metastatic lesions, encompassing both bone and nonregional lymph nodes.

There have been two randomized controlled studies on RT as local therapy for mPC. The first, the STAMPEDE trial, demonstrated that the addition of RT to the primary tumor site in men with newly diagnosed mPC did not improve OS. They reported 3-year survival rates of 62% in the control group and 65% in the RT group; however, the difference was not statistically significant. Moreover, in the subgroup with a low metastatic burden, both OS and failure-free survival were improved in patients who underwent RT (20). Meanwhile, the HORRAD trial, another prospective randomized trial, revealed that the addition of RT to hormone therapy did not lead to improved OS, similar to the findings in the STAMPEDE trial. However, PSA-recurrence-free survival was better in the RT group (12). Furthermore, a recent meta-analysis showed that prostate RT helped to improve failure-free survival and biochemical progression in men with fewer than five bone metastasis (14). Therefore, according to these results, prostate RT is suggested to improve oncological outcomes in omPC. Ultimately, based on these studies, various guidelines recommend combination therapy with ADT alongside androgen receptor axis-targeted agents with/without docetaxel as a primary approach for mPC. Additionally, ADT combined with RT has also been proposed as an option for treating mPC with a low burden.

Regarding studies on prostatectomy, Culp et al.  (6) were the first to report the effect of local therapy on OS and CSS in patients with mPC in a population-based study. They reported that the 5-year OS and predicted CSS were higher in patients undergoing RP (67.4% and 75.8%, respectively) compared to those who did not receive local therapy (22.5% and 48.7%, respectively) (p<0.001). However, a recent multicenter prospective study reported contradictory results. In their study, Buelens et al. showed that RP tended to result in longer castration-resistant cancer-free survival in patients with mPC compared to the standard care group, who initiated treatment with ADT. However, in the multivariable model, RP was not associated with castration-resistant cancer-free survival (HR=0.73, p=0.5) (21). In contrast, a prior study incorporating the “Local Treatment With RP for Newly-diagnosed mPCa” (LoMP) trial registry, focusing on patients who were newly diagnosed with low-volume mPC (defined as the absence of visceral metastasis and three or fewer bone lesions), showed different results (15). They reported that the 2-year CSS (93% vs. 75%, p=0.037) and OS (93% vs. 69%, p=0.007) were better in the RP group than in the “no local therapy” (NLT) group. Moreover, multivariate Cox regression analysis also showed that RP decreased the OS compared to NLT (HR=0.36, p=0.037). In addition, Dai et al. (22) recently assessed the benefits of adding radical local therapy (RLT), predominantly RP, to ADT in omPC in a phase 2 study. With 200 participants, the results showed a 57% reduction in radiographic progression risk and a 56% decrease in mortality risk for the ADT with RLT group over 4 years, highlighting the treatment’s potential to improve survival outcomes. Notably, most studies investigating the therapeutic impact of RP in omPC are based on retrospective analyses. For instance, the prospective study that employed the LoMP registry was not randomized, and encompassed a limited patient cohort. Similarly, the aforementioned phase 2 study, despite its contributions, was limited by its small sample size and the inclusion of patients undergoing treatments beyond RP alone. Therefore, although existing studies have demonstrated the potential for RP to improve treatment outcomes in omPC, most current guidelines do not yet recommend RP for mPC, including cases with a low burden of mPC.

Although some studies have reported that RT and RP each have better oncologic outcomes, studies comparing oncologic outcomes between RP and RT in mPC are rare. For example, Knipper et al.  (16) compared the oncologic outcomes of patients who received RP with those of STAMPEDE arm H patients with a low metastatic burden who received RT. They reported no significant differences in CSS or OS between the two groups. Additionally, Lumen et al.  (15) reported that for select patients with omPC, RP was able to achieve a similar OS and CSS as RT. Similarly, we observed no significant differences in PFS, CSS, or OS between the RP and RT groups in the present study. Moreover, RP and RT did not affect PFS, CSS, or OS in the multivariate analysis.

When considering treatment for the primary tumor in patients with omPC, RT must be administered. However, several problematic situations are encountered when attempting RT in actual clinical practice. For instance, patients with PC undergoing RT need to visit the outpatient clinic daily for a period of 4–6 weeks. For patients residing in regions with limited hospital access, the daily requirement to visit the hospital poses a significant challenge. Due to these reasons, some patients prefer RP over RT, prompting us to compare the oncological outcomes between RP and RT. We believe our results suggest that although RP may not be considered a treatment method in accordance with current guidelines, it could potentially offer a feasible alternative to RT for patients who desire this treatment approach.

Our study had some limitations. First, the diagnosis of omPC in our study was based on CT, bone scans, and PET-CT. Recently, prostate-specific membrane antigen (PSMA)-PET, a more accurate tool for staging prostate cancer, has become increasingly popular. However, at the time when patients included in our study were diagnosed with prostate cancer, PSMA-PET had not yet been introduced in Korea. For this reason, there were no patients in this study who were diagnosed with metastasis using PSMA-PET. Consequently, there is a possibility that some patients who did not meet the actual criteria for omPC were inadvertently included in the study. Additionally, our evaluation of complications was limited to urinary tract complications, without considering other aspects of quality of life such as urinary incontinence or erectile dysfunction after RP or RT. Notably, in cases where there was no significant difference in oncological outcomes between the RT and RP groups, complications following each treatment method may have been be more important for patients when choosing a treatment method. These complications are closely related to quality of life, which, for some patients, is the most important consideration when choosing a treatment modality (23). Another limitation was that our study was based on a retrospective design; therefore, we were unable to control for patient and disease factors, such as age, CCI, initial PSA, biopsy GG, or clinical stage. Additionally, even within the group that received RT, there was variability in treatment modalities, further complicating our analysis. Moreover, the number of patients included in our study was relatively small, making it difficult to generalize our results. Lastly, the impact of additional therapies such as chemotherapy or palliative RT after cancer progression could not be assessed in the multivariate analysis due to the variability in treatment decisions made by physicians based on individual patient circumstances. However, by comparing the oncological results over a relatively long follow-up period compared to those of previous studies, it was possible to confirm the treatment effects of RP and RT over a longer period in omPC.

Currently, RT rather than RP is proposed as one of the treatment options for local therapy in omPC. However, our results demonstrated there were no significant differences in urinary tract complications, PFS, CSS, or OS between the RP and RT groups among patients with omPC. Therefore, RP also showed potential as a treatment option in some patients with omPC. However, due to the limitations present in our study as well as in existing research, more large-scale, well-designed studies are needed before RP can be considered a standard treatment option.

## Data availability statement

The datasets presented in this article are not readily available because exporting data according to institutional policy requires a very strict permission process. Requests to access the datasets should be directed to JK. lumpakcef@yuhs.ac.

## Ethics statement

The studies involving humans were approved by Institutional Review Board of Yongin Severance Hospital. The studies were conducted in accordance with the local legislation and institutional requirements. The ethics committee/institutional review board waived the requirement of written informed consent for participation from the participants or the participants’ legal guardians/next of kin because this was a retrospective study.

## Author contributions

WH: Project administration, Writing – review & editing, Supervision, Resources, Funding acquisition. JP: Writing – review & editing, Methodology, Investigation, Funding acquisition. WJ: Writing – review & editing, Resources, Investigation, Conceptualization. JK: Writing – review & editing, Writing – original draft, Project administration, Methodology, Formal analysis, Conceptualization.

## References

[1] Ryan CJ Elkin EP Small EJ Duchane J Carroll P. Reduced incidence of bony metastasis at initial prostate cancer diagnosis: data from CaPSURE. Urol Oncol. (2006) 24:396–402. doi: 10.1016/j.urolonc.2005.09.003 16962488

[2] Cornford P Bellmunt J Bolla M Briers E De Santis M Gross T. EAU-ESTRO-SIOG guidelines on prostate cancer. Part II: treatment of relapsing, metastatic, and castration-resistant prostate cancer. Eur Urol. (2017) 71:630–42. doi: 10.1016/j.eururo.2016.08.002 27591931

[3] Lowrance WT Breau RH Chou R Chapin BF Crispino T Dreicer R. Advanced prostate cancer: AUA/ASTRO/SUO guideline PART I. J Urol. (2021) 205:14–21. doi: 10.1097/JU.0000000000001375 32960679

[4] Park K Kim JY Park I Shin SH Lee HJ Lee JL. Effectiveness of adding docetaxel to androgen deprivation therapy for metastatic hormone-sensitive prostate cancer in korean real-world practice. Yonsei Med J. (2023) 64:86–93. doi: 10.3349/ymj.2022.0244 36719015 PMC9892544

[5] Oishi T Hatakeyama S Tabata R Fujimori D Fukuda M Shinozaki T. Comparison of neoadjuvant chemohormonal therapy vs. extended pelvic lymph-node dissection in high-risk prostate cancer treated with robot-assisted radical prostatectomy. Sci Rep. (2023) 13:3436. doi: 10.1038/s41598-023-30627-7 36859718 PMC9978020

[6] Culp SH Schellhammer PF Williams MB. Might men diagnosed with metastatic prostate cancer benefit from definitive treatment of the primary tumor? A SEER-based study. Eur Urol. (2014) 65:1058–66. doi: 10.1016/j.eururo.2013.11.012 24290503

[7] Heidenreich A Pfister D Porres D. Cytoreductive radical prostatectomy in patients with prostate cancer and low volume skeletal metastasis: results of a feasibility and case-control study. J Urol. (2015) 193:832–8. doi: 10.1016/j.juro.2014.09.089 25254935

[8] Jang WS Kim MS Jeong WS Chang KD Cho KS Ham WS. Does robot-assisted radical prostatectomy benefit patients with prostate cancer and bone oligometastasis? BJU Int. (2018) 121:225–31. doi: 10.1111/bju.13992 28834084

[9] Cho Y Chang JS Rha KH Hong SJ Choi YD Ham WS. Does radiotherapy for the primary tumor benefit prostate cancer patients with distant metastasis at initial diagnosis? PloS One. (2016) 11:e0147191. doi: 10.1371/journal.pone.0147191 26807740 PMC4726731

[10] Hellman S Weichselbaum RR. Oligometastasis. J Clin Oncol. (1995) 13:8–10. doi: 10.1200/jco.1995.13.1.8 7799047

[11] Weichselbaum RR Hellman S. Oligometastasis revisited. Nat Rev Clin Oncol. (2011) 8:378–82. doi: 10.1038/nrclinonc.2011.44 21423255

[12] Boevé LMS Hulshof M Vis AN Zwinderman AH Twisk JWR Witjes WPJ. Effect on survival of androgen deprivation therapy alone compared to androgen deprivation therapy combined with concurrent radiation therapy to the prostate in patients with primary bone metastatic prostate cancer in a prospective randomised clinical trial: data from the HORRAD trial. Eur Urol. (2019) 75:410–8. doi: 10.1016/j.eururo.2018.09.008 30266309

[13] Parker CC James ND Brawley CD Clarke NW Hoyle AP Ali A. Radiotherapy to the primary tumour for newly diagnosed, metastatic prostate cancer (STAMPEDE): a randomised controlled phase 3 trial. Lancet. (2018) 392:2353–66. doi: 10.1016/s0140-6736(18)32486-3 PMC6269599 30355464

[14] Burdett S Boevé LM Ingleby FC Fisher DJ Rydzewska LH Vale CL. Prostate radiotherapy for metastatic hormone-sensitive prostate cancer: A STOPCAP systematic review and meta-analysis. Eur Urol. (2019) 76:115–24. doi: 10.1016/j.eururo.2019.02.003 PMC6575150 30826218

[15] Lumen N De Bleser E Buelens S Verla W Poelaert F Claeys W. The role of cytoreductive radical prostatectomy in the treatment of newly diagnosed low-volume metastatic prostate cancer. Results from the local treatment of metastatic prostate cancer (LoMP) registry. Eur Urol Open Sci. (2021) 29:68–76. doi: 10.1016/j.euros.2021.05.006 34337536 PMC8317829

[16] Knipper S Beyer B Mandel P Tennstedt P Tilki D Steuber T. Outcome of patients with newly diagnosed prostate cancer with low metastatic burden treated with radical prostatectomy: a comparison to STAMPEDE arm H. World J Urol. (2020) 38:1459–64. doi: 10.1007/s00345-019-02950-0 31511970

[17] Amin MB Edge S Greene F Byrd DR Brookland RK Washington MK. AJCC Cancer Staging Manual. 8 ed. New York, NY: Springer (2017).

[18] Charlson ME Carrozzino D Guidi J Patierno C. Charlson comorbidity index: A critical review of clinimetric properties. Psychother Psychosom. (2022) 91:8–35. doi: 10.1159/000521288 34991091

[19] Soloway MS Hardeman SW Hickey D Raymond J Todd B Soloway S. Stratification of patients with metastatic prostate cancer based on extent of disease on initial bone scan. Cancer. (1988) 61:195–202. doi: 10.1002/1097-0142(19880101)61:1<195::AID-CNCR2820610133>3.0.CO;2-Y 3334948

[20] Ali A Hoyle A Haran ÁM Brawley CD Cook A Amos C. Association of bone metastatic burden with survival benefit from prostate radiotherapy in patients with newly diagnosed metastatic prostate cancer: A secondary analysis of a randomized clinical trial. JAMA Oncol. (2021) 7:555–63. doi: 10.1001/jamaoncol.2020.7857 PMC7893550 33599706

[21] Buelens S Poelaert F Claeys T De Bleser E Dhondt B Verla W. Multicentre, prospective study on local treatment of metastatic prostate cancer (LoMP study). BJU Int. (2022) 129:699–707. doi: 10.1111/bju.15553 34289231

[22] Dai B Zhang S Wan FN Wang HK Zhang JY Wang QF. Combination of androgen deprivation therapy with radical local therapy versus androgen deprivation therapy alone for newly diagnosed oligometastatic prostate cancer: A phase II randomized controlled trial. Eur Urol Oncol. (2022) 5:519–25. doi: 10.1016/j.euo.2022.06.001 35780048

[23] Macdonald H. The importance of quality of life to patient decision making in breast cancer care. Virtual Mentor. (2014) 16:94–7. doi: 10.1001/virtualmentor.2014.16.02.ecas1-1402 24553326

