# Corrigendum: Radical prostatectomy versus radiotherapy as local therapy for primary tumors in patients with oligometastatic prostate cancer

**DOI:** 10.3389/fonc.2024.1427226

**Published:** 2024-06-13

**Authors:** Won Sik Ham, Jee Soo Park, Won Sik Jang, Jongchan Kim

**Affiliations:** ^1^ Department of Urology and Urological Science Institute, Yonsei University College of Medicine, Seoul, Republic of Korea; ^2^ Department of Urology, Yongin Severance Hospital, Yonsei University Health System, Yongin, Republic of Korea

**Keywords:** prostate cancer, oligometastasis, local therapy, radiotherapy, prostatectomy

In the published article, there was an error in the Funding statement. Instead of “faculty research grant from Yonsei University College of Medicine (6-2021-0106)” it should be “new faculty research seed money grant of Yonsei University College of Medicine for 2023 (2023-32-0061)”. The correct Funding statement appears below.

